# Potential toxicity of leachate from the municipal landfill in view of the possibility of their migration to the environment through infiltration into groundwater

**DOI:** 10.1007/s10653-021-00867-5

**Published:** 2021-03-06

**Authors:** Agata Jabłońska-Trypuć, Urszula Wydro, Elżbieta Wołejko, Anna Pietryczuk, Adam Cudowski, Jacek Leszczyński, Joanna Rodziewicz, Wojciech Janczukowicz, Andrzej Butarewicz

**Affiliations:** 1 Department of Chemistry, Biology and Biotechnology, Faculty of Civil Engineering and Environmental Sciences, Białystok University of Technology, Wiejska 45E Street, 15-351 Białystok, Poland; 2 Department of Water Ecology, Faculty of Biology, University of Białystok, Ciołkowskiego 1J Street, 15-245 Białystok, Poland; 3 Department of Environmental Engineering Technology and Systems, Faculty of Civil Engineering and Environmental Sciences, Białystok University of Technology, Wiejska 45E Street, 15-351 Białystok, Poland; 4 Department of Environment Engineering, Faculty of Geoengineering, University of Warmia and Mazury in Olsztyn, Warszawska Street 117a, 10-719 Olsztyn, Poland

**Keywords:** Leachate, Bacteria, Fibroblasts, Melanoma, Cytotoxicity, Apoptosis

## Abstract

Leachate from landfills is a product of complex biological and physicochemical processes occurring during waste storage. In the present study, the toxicity of landfill leachate (LL) to human and bacterial cells was investigated for better understanding of LL environmental toxicity. Studies regarding LL physicochemical properties and cytotoxicity analysis were conducted. In *Escherichia coli*, *Pseudomonas fluorescens*, *Bacillus subtilis*, fibroblasts and melanoma A-375 cells, cell viability assays were applied. For the determination of LL antibacterial activity, twofold dilution series of LL were prepared in the range from 50% to 0.1% (50%, 25%, 12.5%, 6.25%, 3.13%, 1.56%, 0.78%, 0.39%, 0.2%, 0.1%). Human cells viability was examined at LL concentrations of 0.1%, 0.5%, 1%, 1.5%, 2%, 2.5%, 5%, 10%, 15%, 20% and 30%. ROS (reactive oxygen species) content and apoptosis level were also measured in bacterial and human cells under the influence of LL. Unexpectedly obtained results indicate stimulation of bacterial viability by LL. Fibroblasts under the influence of LL showed decrease in their viability and increase in apoptosis level and A-375 melanoma cells showed an increase in relative viability and decrease in apoptosis. ROS level in bacterial cells was elevated in higher LL concentrations and decreased in lower LL concentrations. In human cells, ROS content was rather high in both tested cell lines. Presented results indicate cytotoxic potential of analyzed LL and the necessity of LL monitoring because it may pose a health hazard for exposed human populations and the whole human environment.

## Introduction

Proper waste management is a key aspect in both developed and developing countries. Landfill disposal is one of the oldest waste management methods currently in use, but it is still very common worldwide. During storage, the waste undergoes many complex biological and physicochemical processes resulting in a product called leachate. In the composition of such a product, the basic chemical categories of compounds from the groups of important environmental pollutants are identified. The chemical composition of the leachate depends mainly on the type of waste stored in the landfill, but also on the degree of water infiltration, the degree of residual hydration, technology used on the landfill and the degree of waste degradation (Christensen et al. 2001; Torretta et al. 2017; Yao 2017). Leachate from landfills constitutes an important aspect of environmental pollution because of its ability to the penetration into groundwater, migration over considerable distances and accumulation in various links in the food chain, therefore posing a threat to the ecosystem and public health. However, a landfill releases a wide range of chemicals resulting from the degradation of waste not only in the form of leachate. It can also release hazardous pollutants in the form of gases and solid particles, especially if it is an active landfill. Gases and particles in the air above a landfill can get into the respiratory tract of people in the vicinity of the landfill or those working on its premises. A health risk may even occur after accidental exposure in an uncontrolled dumpsite. Another possibility for human exposure to leachates is their migration through groundwater to bodies of water such as ponds and lakes used for recreational purposes (Khalil et al. 2018; Mishra et al. 2018; Naveen et al. 2017; Przydatek 2019; Sang and Li 2004).

Traditional methods of landfill leachate (LL) analysis include primarily chemical tests involving the determination of heavy metals and organic compounds with carcinogenic, estrogenic and toxic properties. These methods allow for a preliminary estimation of danger and risk that LL poses to the environment and human (Clarke et al. 2015). However, only tests based on biological model organisms allow to examine the response to all, both chemical and biological components of leachate and thus assess their potential toxicity to living organisms. If the biological model is chosen appropriately or when the tests are carried out on several different biological models, then there is a chance to obtain representative toxicity results of the complex matrix such as leachate. It is possible to study the bioavailability and interaction of sample components and, as a consequence, additive, synergistic or antagonistic effects in a given biological model (Farre and Barcelo 2003). Ghosh et al. in their review work present various methods for toxicological analysis of complex matrices containing various environmental pollutants in the form of mixtures of compounds (Ghosh et al. 2017). In order to study the potentially toxic effects of leachate components on living organisms, mammalian cell lines, fish cell lines, bacterial strains and selected algae species are being used as biological models. Baderna et al. in their review article focus on human cell cultures in vitro as the main biological model allowing to estimate the potential risk posed by LL on human health (Baderna et al. 2019). One of the main reasons for the growing use of biological tests based on in vitro human cell cultures is their high sensitivity to mutagenic and carcinogenic toxins present in environmental matrices. Complexed mixture such as leachate exhibits its genotoxic and/or carcinogenic potential only after metabolic activation, therefore metabolic competence of human cell lines makes them an ideal model for cytotoxicity testing (Glatt et al. 1990). Many different cell lines are being used for the testing of LL toxicity, e.g.: MFC-7, lymphocytes, fibroblasts, Jurkat line, CHO, HOS, NIH/3T3, HepG2, melanoma cell lines and MVLN among others. The most frequently used assays are MTT assay, comet assay and Trypan blue exclusion assay. The most important measurement methods focus on measuring cytotoxicity, proliferation, cell viability and genotoxicity. Usually, the average time of exposure to the tested leachate in a wide range of percentage concentrations is from 24 to 48 h (Baderna et al. 2019). However, to verify the obtained results of cytotoxicity tests, several tests based on different principles should be performed, e.g., cell membrane integrity test (LDH test and neutral red test), test of activity of selected caspases as markers of apoptosis, and test of energy metabolism disorders (level ATP, reduction of resasurin) (Prasse et al. 2015).

The aim of the study was to determine potential toxicity of leachate from the local municipal landfill considering the possibility of its eventual migration to the environment by infiltration into the groundwater (Sang and Li 2004). For this purpose, we analyzed its chemical composition and its activity in different biological models. Nevertheless, only a few studies have investigated the cytotoxic effect of landfill leachate especially in human cells, which is why we chose to examine bacterial and human cells viability, apoptosis and ROS content. So the present study focuses mainly on the most appropriate research model—bacterial strains and human cell lines. Concentration range was selected for the experiment on the basis of literature data (Bakare et al. 2007). Moreover, the aim of this work was also presentation of the variety of methods mainly based on different biological models, dedicated for leachate toxicity testing.

## Materials and methods

### Sample processing

**Fig. 1:**
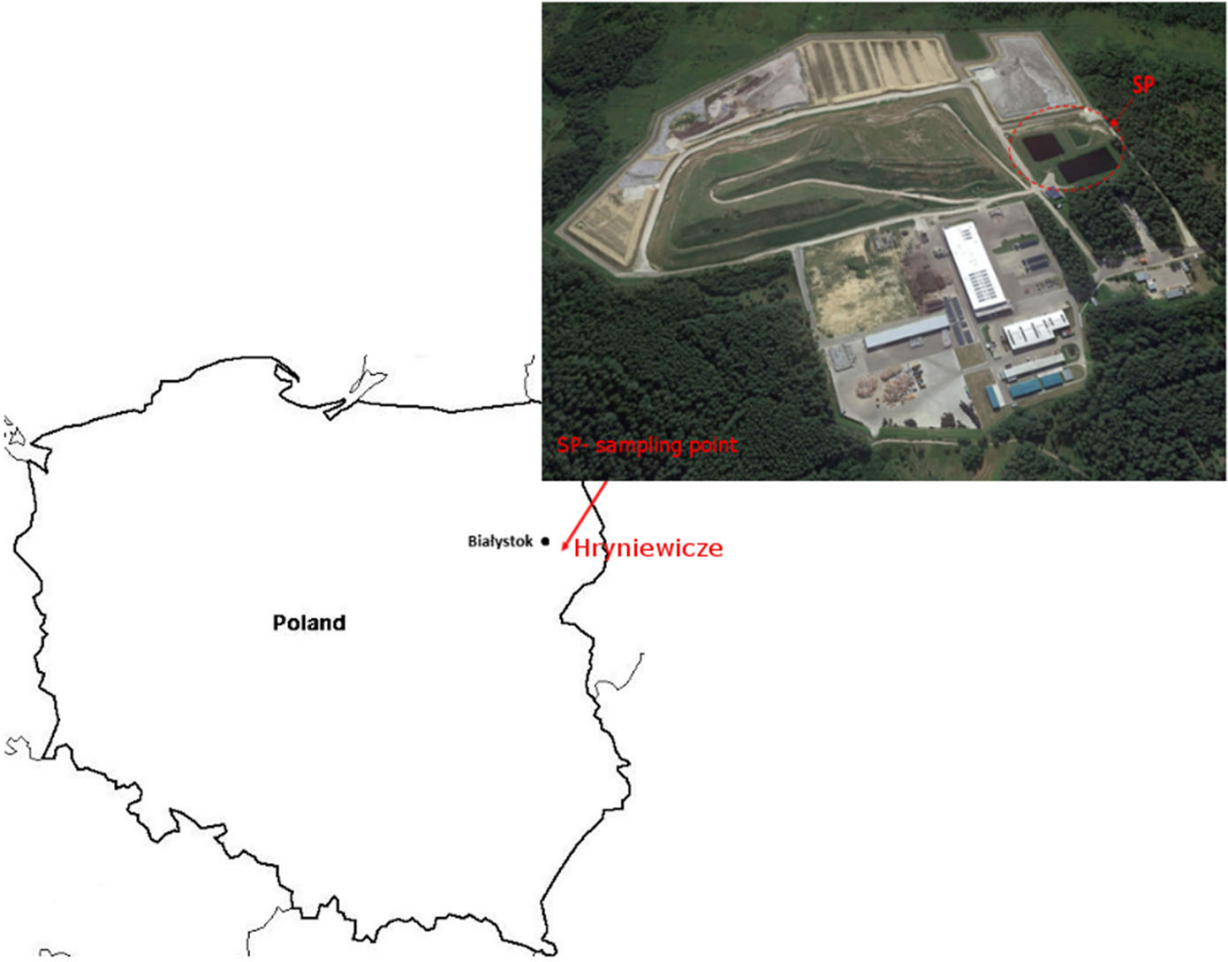
Landfill sites of interest in the northeastern part of Poland (Hryniewicze)

In the present study, leachate was obtained from many points of the surface and from the different depths of retention basins in solid waste landfill located in the northeastern part of Poland (Hryniewicze) (Fig. 1). A number of 50 samples were collected from different depths and from different locations within the retention basins. Subsequently, the samples were combined and mixed, and three samples were taken from one representative group sample and used for testing to perform the analysis in triplicate. All samples were collected in special plastic bottles with minimal headspace and filtered through mixed cellulose ester 0.45 µm membranes. Subsequently, leachate was filtered through the membranes with 0.22 µm porosity three times in order to prevent microbiological contamination of A-375 melanoma cells, fibroblast cells and bacterial cells. The obtained aqueous extracts from each sample were used for further analysis. LL samples were stored in a refrigerator at temperature 4 °C in the dark.

### Landfill leachate characterization, physicochemical properties

The concentrations of total organic carbon (TOC) as well as total nitrogen (TN) were determined in the laboratory using a high-temperature catalytic combustion method in a Shimadzu TOC-5050A analyzer. Heavy metals: Hg, Pb, Cd, Cr, Ni, Cu, Zn and Fe, as well as aluminum, sulfur, phosphorus and tin, were analyzed on a TXRF (Total X-Ray Reflection Fluorescence) S2 PICOFOX apparatus, Bruker, Berlin, Germany (Mages et al. 2003). Biochemical oxygen demand (BOD) was determined with the OxiTop measurement system. Chemical oxygen demand (COD) was measured using COD cell test according to manufacturer’s instruction (Merck Millipore). The pH-meter Hach session 4 was used to determine the pH and electrical conductivity (EC). The turbidity was determined by the nephelometric method with the WTW 550 IR device. The color was determined on a platinum-cobalt scale using a Hach DR 4000 spectrophotometer. The Thermo Scientific ICS 5000 + ion chromatograph was used to determine the content of inorganic ions in the crude effluents. PAHs were determined applying the GC–MS system Triple Quad 7890B according to ISO 18,287:2008 (PN-ISO 18,287:2008). The study included 16 specific PAHs: naphthalene, acenaphthylene, acenaphthene, fluorine, phenanthrene, anthracene, fluoranthene, pyrene, benzo (a) anthracene, chrysene, benzo (b) fluoranthene, benzo (k) fluoranthene, benzo (a) pyrene, indeno (1, 2, 3-cd) pyrene, dibenzo(a,h)anthracene, benzo(g, h, i)perylene.

### Cytotoxicity of LL and ROS content in bacteria cells

#### Microbial strains

*Escherichia coli* (ATCC 25,922), *Pseudomonas fluorescens* (ATCC 13,525) and *Bacillus subtilis* (ATCC 6633) were obtained from the American Type Culture Collection (Manassas, VA, USA). Bacterial strains were selected because of their designation for the environmental pollutants studies (Zhong et al., 2016). Studied strains of bacteria were grown overnight in Mueller Hinton II Broth at 37 °C (*E. coli*, *B. subtilis*) and at 26 °C (*P. fluorescens*). MH II Broth is a special medium dedicated for toxicity testing. The composition of the medium per 1000 ml with a pH—7.4 ± 0.2 was beef extract 2.0 g, casein hydrolysate 17.5 g and starch 1.5 g. Then, the overnight cultures were diluted in fresh MH II Broth in order to obtain 10^8^ CFU/mL (CFU – colony forming units). For the analysis of the antimicrobial activity of LL, 10^6^ CFU/mL inoculum of *E. coli, P. fluorescens* and *B. subtilis* cells were used.

#### Determination of antibacterial activity

Twofold dilution series of LL were prepared according to Jabłońska-Trypuć et al. (2019a). The final concentrations of LL in each well were 50%, 25%, 12.5%, 6.25%, 3.13%, 1.56%, 0.78%, 0.39%, 0.20% and 0.10%. Antimicrobial activity of LL against *E. coli, P. fluorescens* and *B. subtilis* was estimated using luminescence method (BacTiter-Glo™) and MTT assay. The details were described previously (Jabłońska-Trypuć et al. 2019b; 2020). The antimicrobial activity of tested LL was presented as a relative cell viability of *E. coli*, *P. fluorescens* and *B. subtilis* as compared to control untreated cells and expressed in percentage. The determination of antimicrobial activity in all samples was conducted in triplicates.

#### Estimation of ROS level

*B. subtilis, E. coli* and *P. fluorescens* cells were grown overnight in fresh MH II Broth. After 24 h of growth, cells were treated with LL (concentrations: 50%, 25%, 12.5%, 6.25%, 3.13%, 1.56%, 0.78%, 0.39%, 0.20% and 0.10%), and incubation was continued for 24 h. At this time, H2DCFDA (2′,7′-dichlorodihydrofluorescein diacetate) was added to a final concentration of 9.6 μM and incubation was continued in the dark. Fluorescence was measured in a GloMax™ Multi Detection System (Promega) plate reader (excitation, 495 nm; emission, 527 nm). Control cells were incubated without tested LL. ROS level was estimated as relative H2DCFDA fluorescence (Maynard et al. 2019). All the measurements were conducted in triplicates.

### Cytotoxicity of LL, apoptosis and ROS content in human cell culture

#### Human cell lines

The influence of LL was studied in skin cancer melanoma A-375 cell line and normal human skin fibroblasts, which both were obtained from American Type Culture Collection (ATCC). Melanoma cells and fibroblasts were cultured in DMEM containing glucose at 4.5 mg/ml (25 mM) (PAN Biotech, Germany) supplemented with 10% FBS (PAN Biotech), penicillin (100U/mL, PAN Biotech) and streptomycin (100 μg/mL, PAN Biotech) at 37 °C in a humified atmosphere of 5% CO_2_ in air. A fetal bovine serum (FBS) concentration of 10% is recommended by the ATCC.

The cells viability in both tested cell lines was examined at LL concentrations of 0.1%, 0.5%, 1%, 1.5%, 2%, 2.5%, 5%, 10%, 15%, 20% and 30%. The incubation times were 24 h and 48 h.

#### Estimation of tested LL Cytotoxicity

The compounds were added to the cultured cells for a final LL concentration in the range of 0.1%, 0.5%, 1%, 1.5%, 2%, 2.5%, 5%, 10%, 15%, 20% and 30% of LL. The control cells were incubated without the test compounds.

LL cytotoxicity was measured by using two methods. One of them is method of Carmichael using 3-(4,5-dimethylthiazol-2-yl)-2,5-diphenyltetrazolium bromide (MTT, Sigma-Aldrich) (Carmichael et al. 1987). Fibroblast cells were seeded in 96-well plate at a density of 2 × 10^4^ cells/well. Melanoma cells were seeded in 96-well plate at a density of 5 × 10^3^ cells/well. Cells cultured for 24 h and 48 h were treated with LL. After 24 h and 48 h, cells were washed three times with PBS and subsequently incubated with 10µL of MTT solution (5 mg/mL in PBS) for 2 h at 37 °C in 5% CO_2_ in an incubator. Subsequently, 100µL of DMSO was added and cells were incubated in the dark for the next 2 h. The absorbance was measured at 570 nm in a microplate plate reader GloMax^®^-Multi Microplate Multimode Reader. The viability of fibroblast cells and melanoma cells was calculated as a percentage of control cells, incubated without tested compound. All the experiments were done in triplicates.

The second method was based on luminescence measurements. To measure LL cytotoxicity, CellTiter-Glo^™^ 2.0 Assay (Promega, Madison, WI) was used. The measurement was conducted according to manufacturer’s protocol. Luminescence was measured with a GloMax^®^-Multi Microplate Multimode Reader. The study was performed in triplicate taken to ensure consistent results were obtained.

#### Estimation of apoptosis

Apoptosis was estimated in both cell lines by using bioluminescent test based on caspase 3/7 activity measurement. Cells from both analyzed lines were seeded in 96-well white plates and cultured for 24 h. After that time, cells were subjected to presented range of concentrations of LL for 24 and 48 h. After a specified incubation time, medium was removed and Caspase-Glo^®^ 3/7 assay was conducted according to the manufacturer’s instructions. The assay is based on the measurements of the luminescence level, which is proportional to the activity of caspases in analyzed cell lines. GloMax^®^-Multi Microplate Multimode Reader was used in order to measure luminescent signal in samples. The study was performed in triplicate taken to ensure consistent results were obtained.

The average level of apoptosis was also estimated by using fluorescent microscopy method with the application of propidium iodide and calcein-AM dyes (Cell stain double staining kit containing propidium iodide and calcein—AM, Sigma-Aldrich, St. Louis, MO, USA).

#### Intracellular ROS Detection

Intracellular reactive oxygen species (ROS) level was examined at LL concentration range from 0.1 to 30% after 24 h and 48 h of incubation. It was determined using dichlorodihydrofluorescein diacetate (DCFH-DA), (Sigma, St. Louis, MO, USA) according to Krętowski, et al. (2017). Method is based on the reaction catalyzed by cellular esterases giving non-fluorescent compound, which is subsequently oxidized by intracellular ROS into a fluorescent 2′,7′–dichlorofluorescein (DCF).

Fibroblasts were seeded at a density of 2 × 10^4^ cells/well and melanoma cells at a density of 5 × 10^3^cells/well, in 96-well black plates. After 24 h, the medium was removed, the cells were stained with 10 μM of DCFH-DA in PBS at 37 °C, 5% CO_2_ incubator, for 45 min. Next, the dye was removed and replaced with LL resuspended in culture medium in presented range of concentrations for both cell lines. The cells were cultured for 24 h and 48 h. Then, the DCF fluorescence intensity was measured by using the GloMax^®^-Multi Detection System at the excitation wavelength of 485 nm and the emission wavelength of 535 nm. The intracellular ROS generation in fibroblast and melanoma cells was shown as the intensity of fluorescence of the DCF.

### Statistical analysis

All data are given as mean values from three measurements ± SD (standard deviation). Differences between treatments and untreated control human cells were analyzed by one-way ANOVA, followed by Dunnett’s procedure for multiple comparisons. Significant effects are represented by *p* ≤ 0.05 (*), *p*  ≤ 0.01 (**), *p*  ≤ 0.001 (***). To compare the means for treatments and tested bacteria strains, two-way analysis of variance (ANOVA) followed Tukey test was applied. Significance was considered when *p*  ≤ 0.05. The connections between different studied variables determined by PCA (principal components analysis) which is based on Pearson’s correlation and was presented as biplot graph. Statistica 13.0 (StatSoft, Kraków, Poland) was used.

## Results

### Physicochemical properties of landfill leachates

The main physicochemical properties of LL samples and filtrates obtained from leachate after two-step filtration are presented in Table 1. Among all analyzed metals, the highest values were observed for Fe, Sn and Al. Regarding studied ions, the highest values were obtained for Cl^−^, K^+^ and NH_4_^+^. In the group of PAHs, the highest values were obtained for dibenzo[a,h]anthracene, acenaphthene and benzo[a]anthracen. After filtration, mainly, decreases in chemical parameters were noticed. Exceptions are Zn and Cd, where rather low increases were obtained.

**Table 1:** Physicochemical properties of LL. Each value in the table is the mean of triplicates

Parameter	RL	FL
pH	7.8	7.5
EC (mS/cm)	10.62	9.48
Color (Pt–Co units)	2710	1951
Turbidity (NTU)	16	9
TOC (mg/L)	592	510
BOD (mg/L)	64	55
COD (mg/L)	1900	1410
TN (mg/L)	335.5	322
K^+^ (mg/L)	859.725	754.802
NH_4_^+^(mg/L)	583.18	571.22
P (mg/L)	31.042	21.083
Ca^2+^(mg/L)	155.311	109.113
Mg^2+^ (mg/L)	38.783	37.288312
Cl^−^ (mg/L)	1561.084	1300.817
S (mg/L)	164.345	117.208
Fe (mg/L)	5.611	4.774
Mn (mg/L)	0.282	0.225
Cu (mg/L)	0.089	0.082
Ni (mg/L)	0.166	0.134
Cr (mg/L)	0.611	0.056
Zn (mg/L)	0.194	0.212
Pb (mg/L)	0.000044	0.000036
Cd (mg/L)	0.01	0.014
Sn (mg/L)	18.779	12.756
Al (mg/L)	3.003	0.035
Hg (mg/L)	< 0.001	< 0.001
Naphthalene (µg/L)	0.343	0.119
Acenaphthalene (µg/L)	0.061	0.010
Acenaphthene (µg/L)	0.394	0.018
Fluorene (µg/L)	0.087	0.016
Phenanthrene (µg/L)	0.365	0.099
Anthracene (µg/L)	0.116	0.022
Fluoranthene (µg/L)	0.429	0.331
Pyrene (µg/L)	0.293	0.351
Benzo[a]anthracen (µg/L)	0.494	0.088
Chrysene (µg/L)	0.550	0.345
Benzo[b]fluoranthene (µg/L)	0.725	0.799
Benzo[k]fluoranthene (µg/L)	0.615	0.509
Benzo[a]pyrene (µg/L)	0.223	0.254
Indeno[1,2,3-cd]pyrene (µg/L)	0.192	0.135
Dibenzo[a,h]anthracene (µg/L)	0.503	0.079
Benzo[ghi]perylene (µg/L)	0.321	0.335

*RL* Raw leachate, *FL* Filtrates from leachate

### Cytotoxicity of LL and ROS content in bacteria cell

**Fig. 2:**
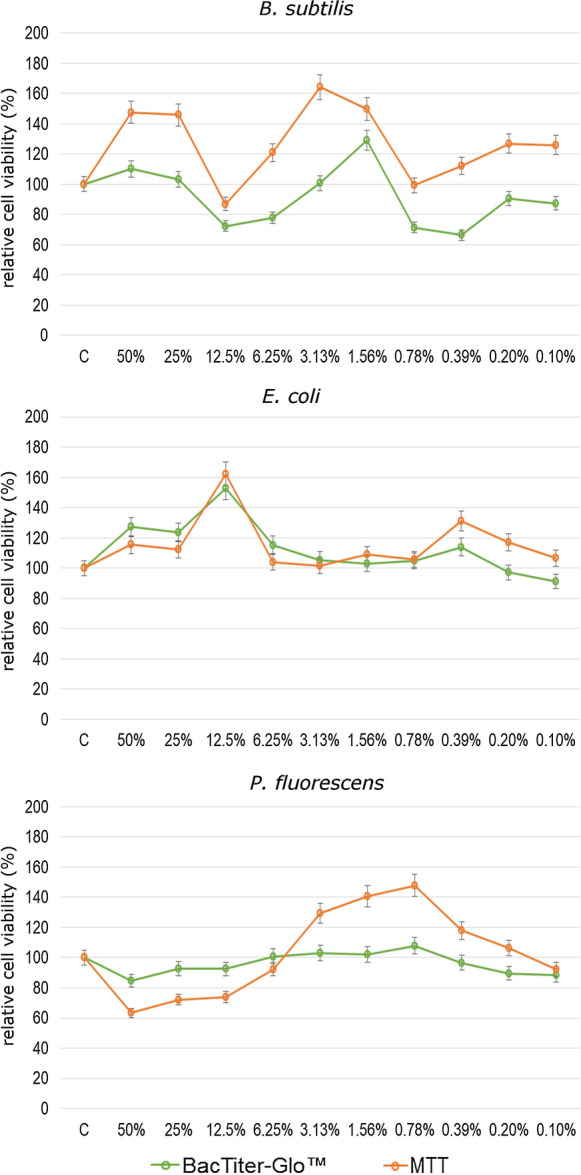
Antimicrobial activity of LL at 50%, 25%, 12.5%, 6.25%, 3.13%, 1.56%, 0.78%, 0.39%, 0.20% and 0.10% concentrations against *B. subtilis*, *E. coli* and *P. fluorescens* presented as relative cell viability [%] and measured by BacTiter-Glo^™^ and MTT methods. Data are presented as mean of triplicates ± SD

**Fig. 3:**
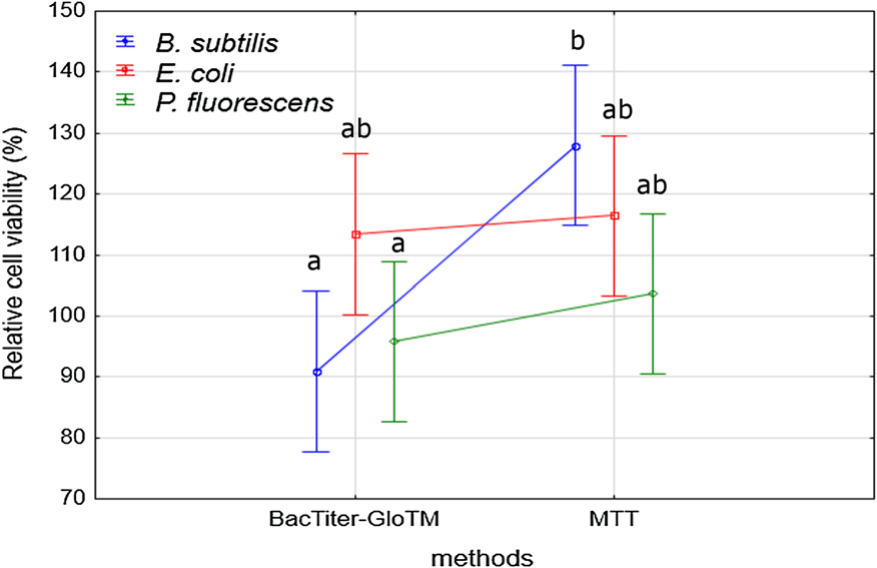
Antibacterial activity of LL depending on studied bacterial strain (*B. subtilis*, *E. coli* and *P. fluorescens*) and used method (BacTiter-Glo^™^ and MTT). The same letters above the bars indicate no statistically significant differences between means from triplicates evaluated by Tukey’s test at *p* < 0.05

**Fig. 4:**
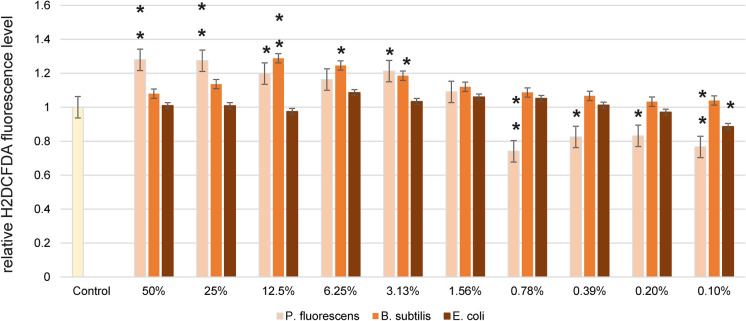
ROS content in *B. subtilis*, *E. coli* and *P. fluorescens* exposed to different concentrations of LL for 24 h expressed as a relative H2DCFDA fluorescence level. Each value on the graph is the mean of three measurements and error bars show the standard deviation (SD). *p < 0.05, **p  < 0.01 and ***p  < 0.001 represent significant effects between treatments and control followed by a Dunnett’s test

Figures 2 and 3 show the effect of LL in different concentrations on the growth of *B. subtilis, E.coli* and *P. fluorescence* strains, which was measured by using two methods: BacTiter-Glo^™^ based on luminescence measurement and colorimetric method with MTT reagent. The results obtained for *B. subtilis* show that the highest stimulatory effect was observed after application of LL in the concentration of 1.56% (BacTiter-Glo^™^) and 3.13% (MTT) and the bacterial growth was higher by about 24% (BacTiter-Glo^™^) and 64% as compared to control. According to the results obtained by using BacTiter-Glo™ assay after application of LL at 12.5%, 6.25%, 0.78%, 0.39%, 0.20% and 0.10% concentrations, a decrease in *B. subtilis* viability from 71 to 90% as compared to control was noticed. By using MTT assay, in most of the tested LL concentrations, the stimulatory effect was observed. Leachate only at 3.13% concentration caused growth inhibitory effect. The results of multi-variance analysis on the relative cell viability of *B. subtilis* showed that the significantly higher values of the tested parameter were obtained for MTT assay. Regarding the influence of LL on viability of *E. coli*, we observed that each of tested concentration caused an increase in cell viability in both used method. The highest stimulatory effect was noted at 12.5% of LL. Results obtained for *P. fluorescens* showed that the range of LL concentrations from 6.25% to 0.78% (BacTiter-Glo^™^) and from 3.13 to 0.20% (MTT) caused an increase in cell viability as compared to control. The highest values were observed at 0.78% for both methods. The highest decrease in viability of *P. fluorescens* was observed for LL concentration of 50%. Non-significant differences between relative cell viability of tested bacterial strains measured by two independent methods were observed. The significant differences were obtained only for *B. subtilis* depending on method used. One of the most likely explanation of changes in cells viability observed under the influence of LL is an increase in oxidative stress level. Therefore, we conducted experiments regarding reactive oxygen species generation in *B. subtilis, E.coli* and *P. fluorescence* strains growing in the presence of LL (Fig. 4). Chemically reduced derivative of fluorescein–2′,7′-dichlorodihydrofluorescein diacetate was used in order to measure ROS content. An increase in ROS level presented in Fig. 4 was correlated with the decrease in cell viability. The highest obtained values were estimated for the highest LL concentrations. However, lower concentrations of tested LL, such as 0.1%, 0.2%, o.39% and 0.78%, caused statistically significant decreases in analyzed parameter (at *p*  < 0.05 and *p*  < 0.01).

### Cytotoxicity, apoptosis and ROS content in human cell lines

**Fig. 5:**
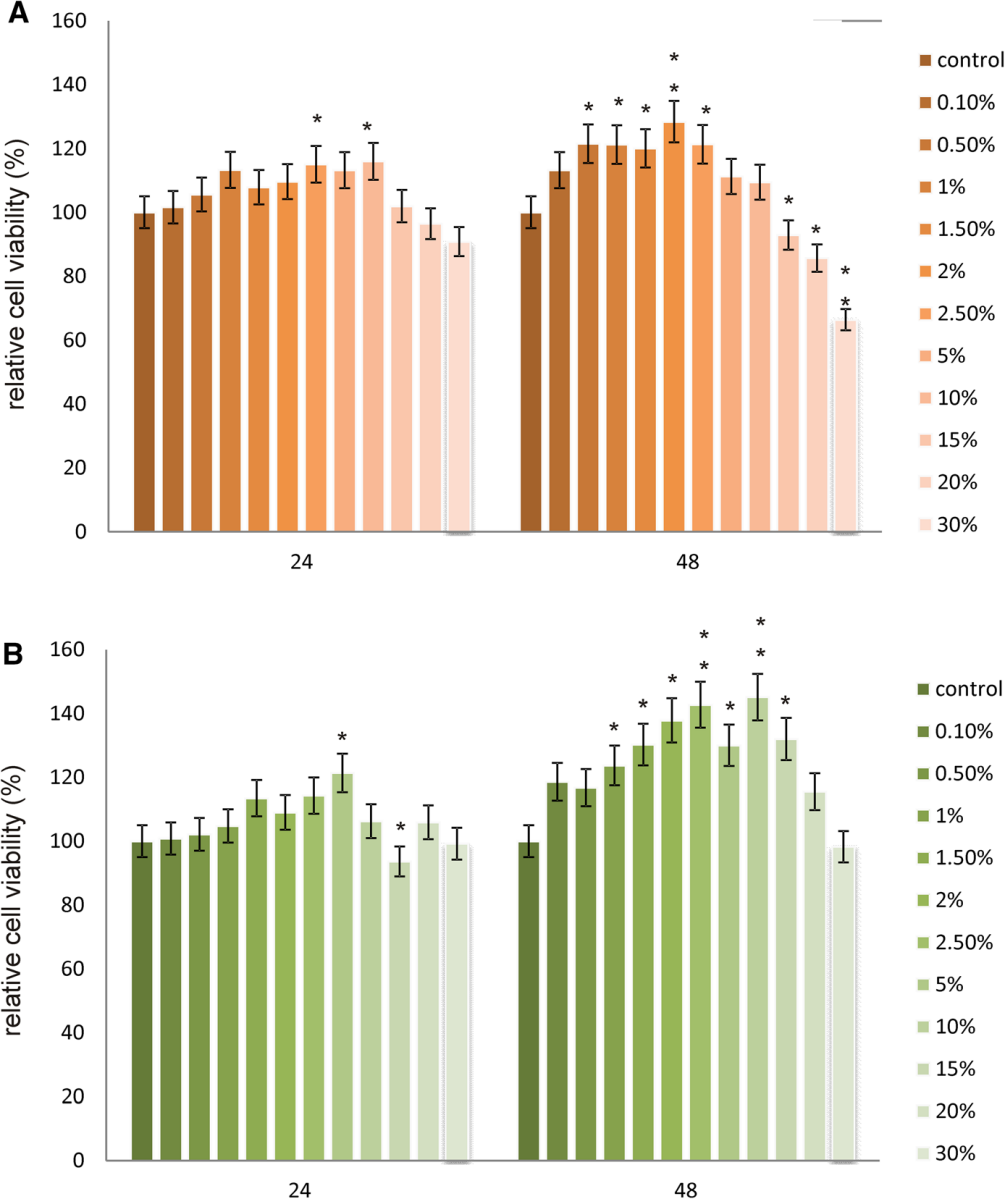
Relative viability of A-375 melanoma cells exposed to different concentrations of LL for 24 and 48 h calculated as a percentage of control cells measured by using **a** CellTiter-Glo^™^ and **b** MTT assay. Each value on the graph is the mean of three measurements and error bars show the standard deviation (SD). *p  < 0.05, **p  < 0.01 and ***p  < 0.001 represent significant effects between treatments and control followed by a Dunnett’s test

**Fig. 6:**
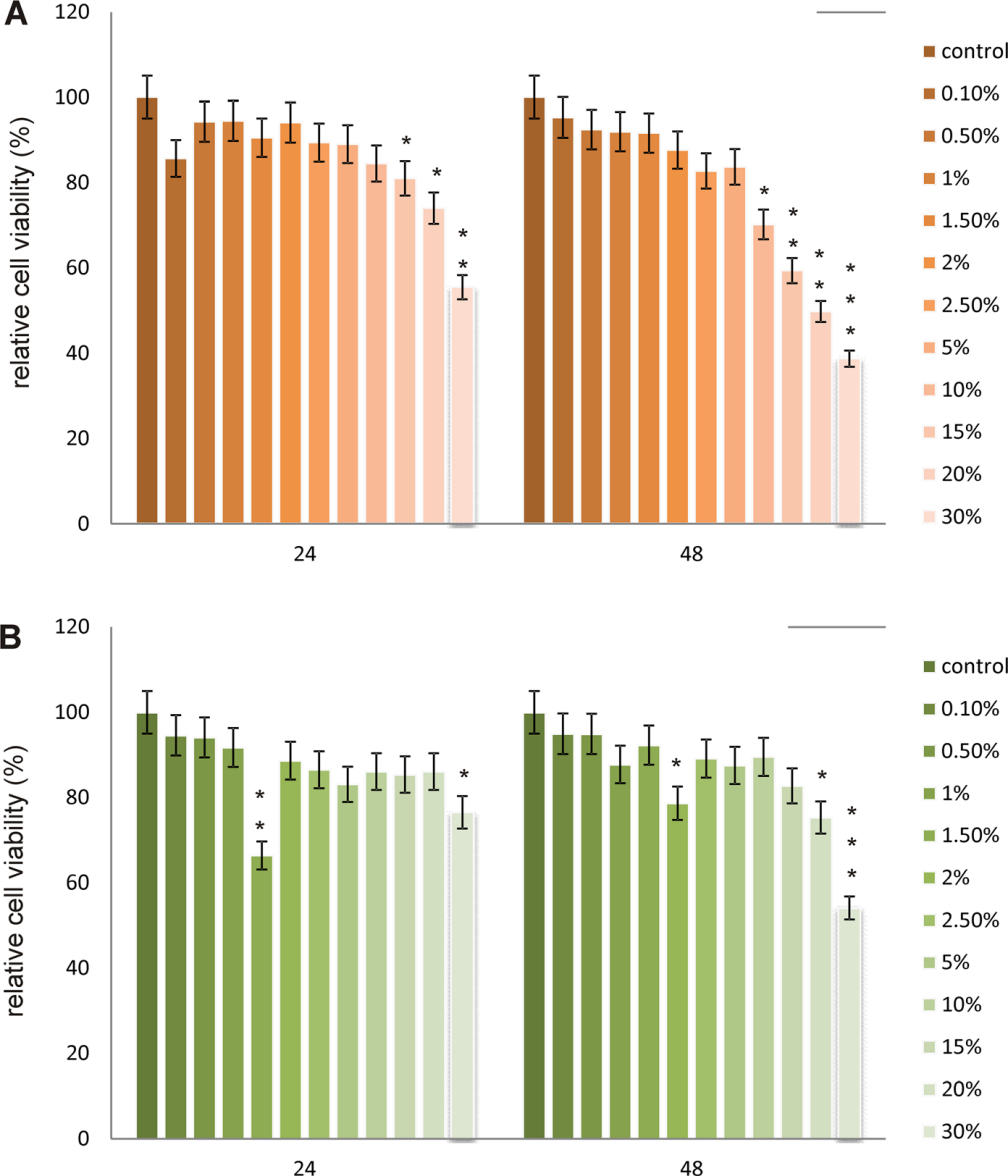
Relative viability of skin fibroblast cells exposed to different concentrations of LL for 24 and 48 h calculated as a percentage of control cells measured by using **a** CellTiter-Glo^™^ and **b** MTT assay. Each value on the graph is the mean of three measurements and error bars show the standard deviation (SD). *p < 0.05, **p < 0.01 and ***p < 0.001 represent significant effects between treatments and control followed by a Dunnett’s test

In both analyzed cell lines, different results regarding relative cell viability were observed (Figs. 5and 6). MTT assay results revealed that A-375 melanoma cells viability was significantly enhanced under the influence of tested LL, especially after 48 h incubation. The highest increases were observed at 2.5%, 5% and 10% concentrations reaching 142%, 130% and 145% as compared to control untreated cells expressed as 100% of relative viability. Luminescent CellTiter-Glo assay gave similar results, indicating significant increases in melanoma cells viability after 48 h incubation. The most significant changes in analyzed parameter were observed in similar concentration range, at 2%, 2.5% and 5% concentration of filtrate used. On the other hand, as it could have been expected, in fibroblasts cell line, a significant decreases in cell proliferation under the influence of every studied LL concentration were noticed. In both incubation times as the LL concentration increased, decreases in cell viability were noted. The results obtained in the MTT test were confirmed in luminescent tests.

**Fig. 7:**
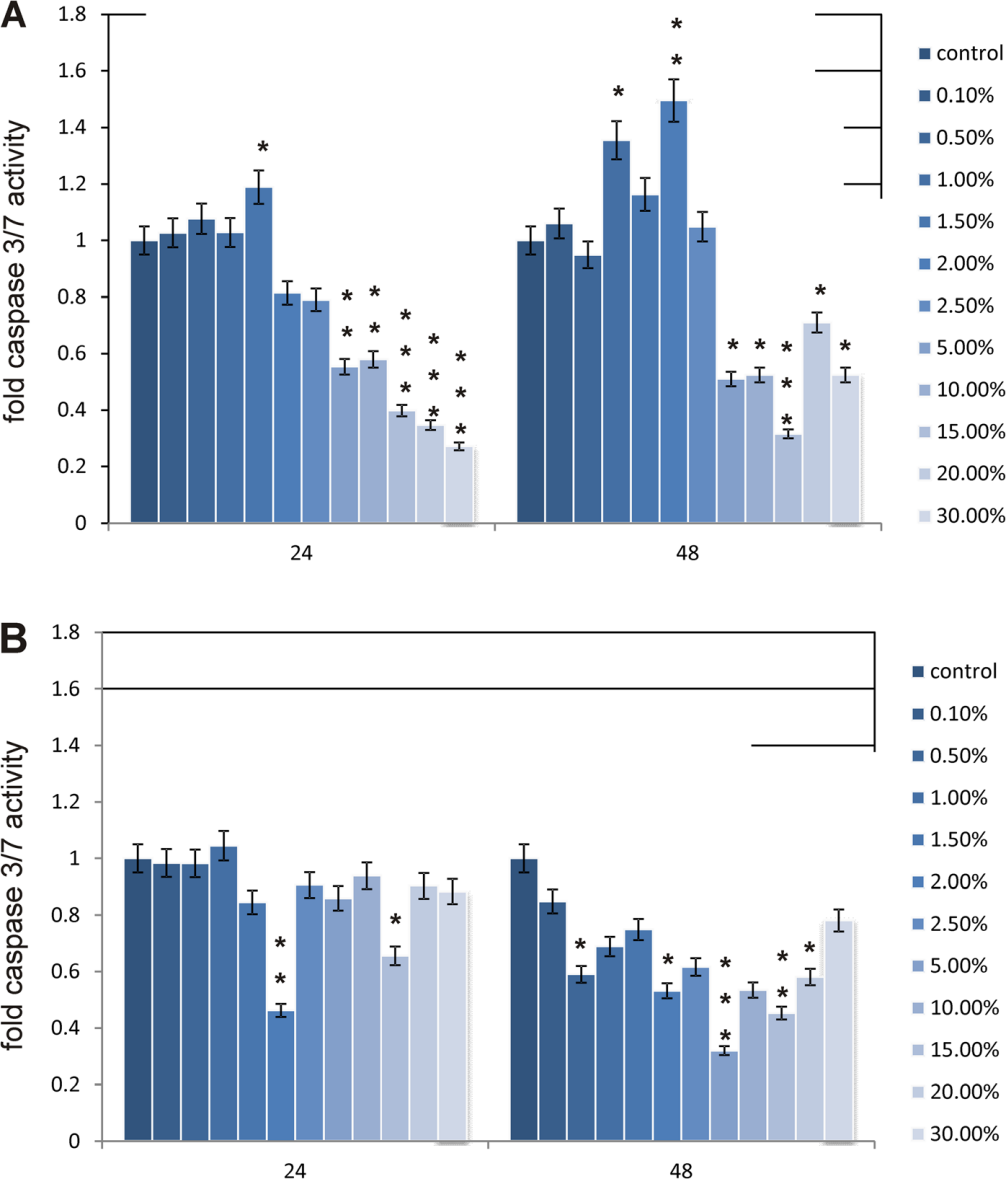
Apoptosis in (**a**) fibroblast cells and (**b**) A-375 melanoma cells exposed to different concentrations of LL for 24 h and 48 h expressed as a fold of caspase 3/7 activity. Each value on the graph is the mean of three measurements and error bars show the standard deviation (SD). *p< 0.05, **p < 0.01 and ***p< 0.001 represent significant effects between treatments and control followed by a Dunnett’s test

**Fig. 8:**
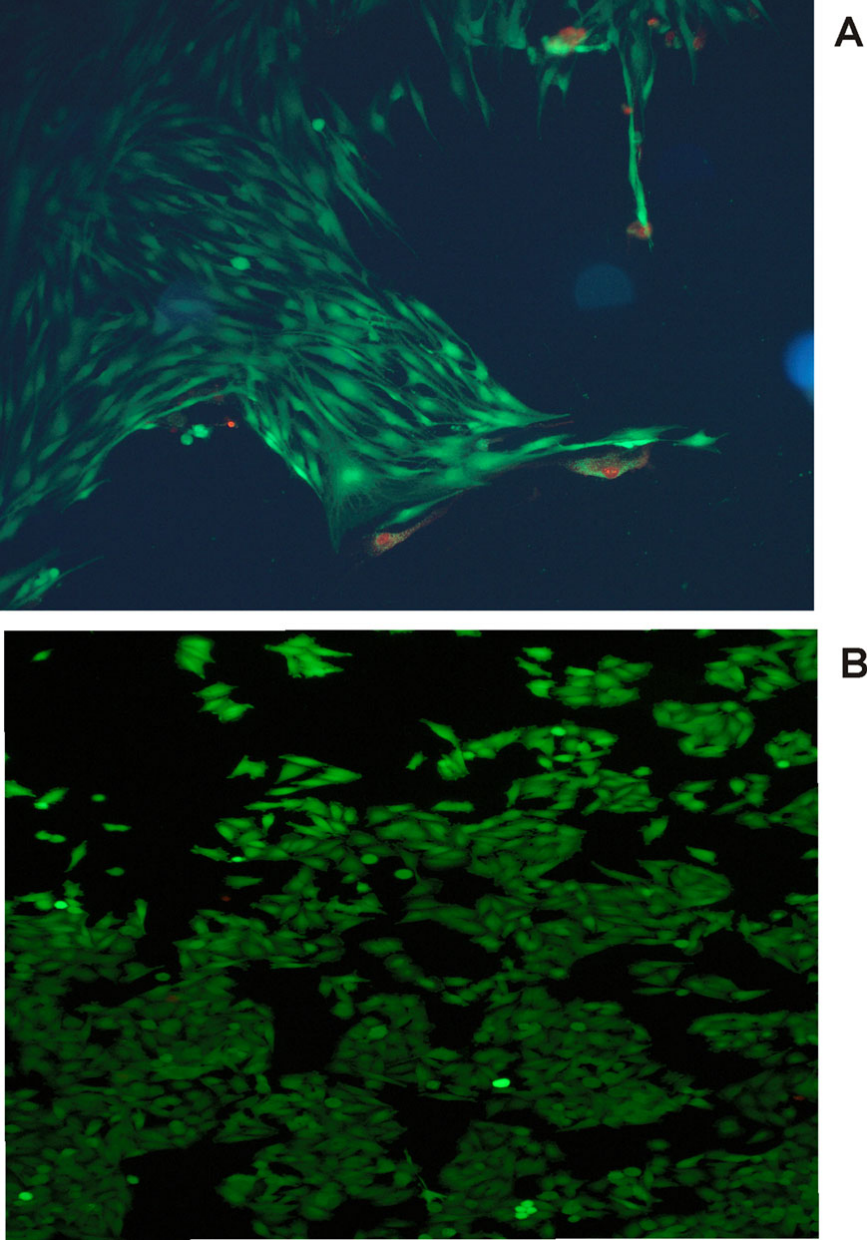
The effect of selected concentrations of LL on apoptosis in (**a**) fibroblast cells and (**b**) melanoma cells evaluated by fluorescence microscope assay (200 × magnification). The cells were incubated with 2% of LL—fibroblasts and 5% of LL—melanoma cells for 48 h and stained with Calcein-AM and propidium iodide. We present representative images form one of three independent experiments

**Fig. 9:**
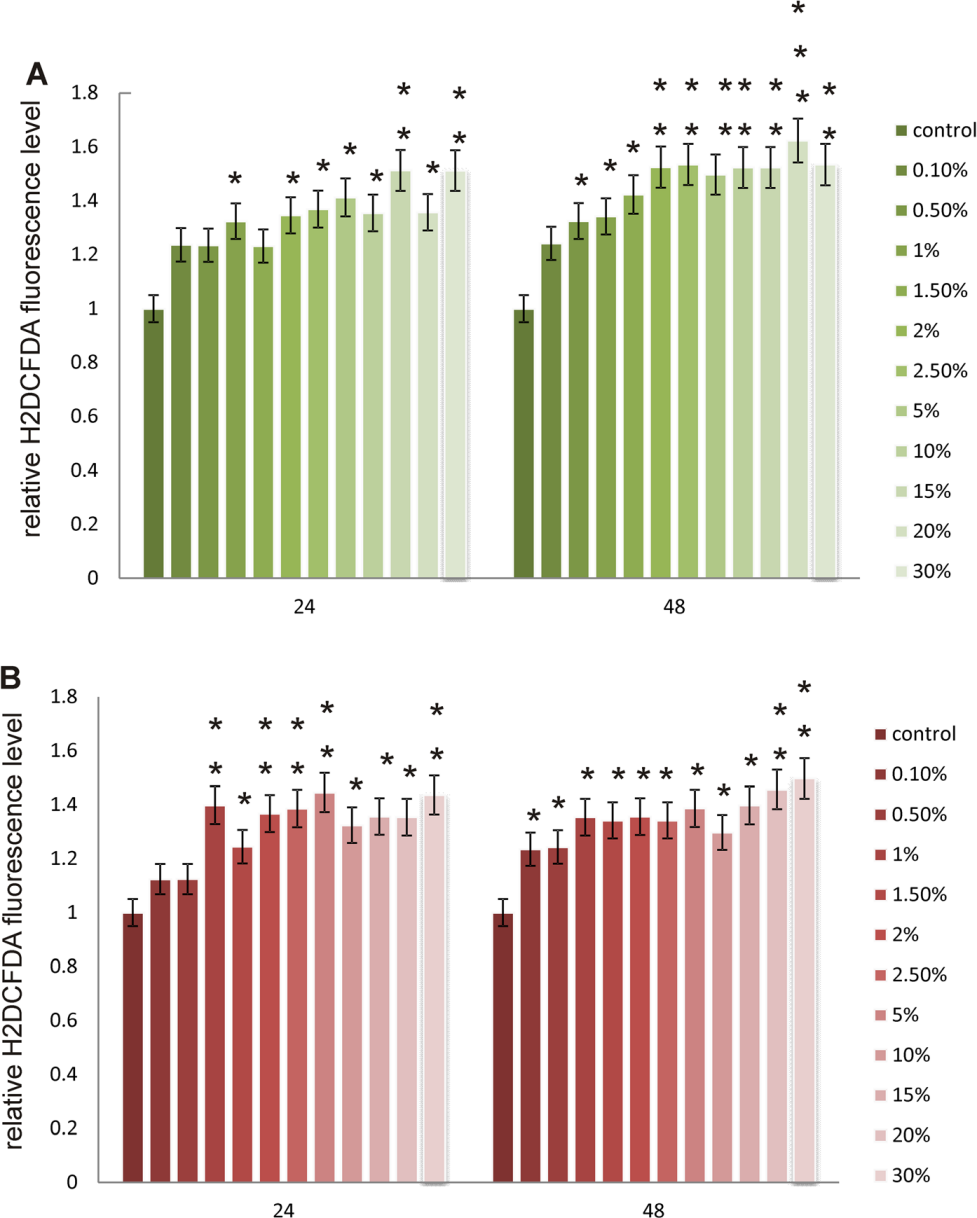
ROS content in fibroblasts (**a**) and melanoma cells (**b**) exposed to different concentrations of LL for 24 and 48 h expressed as a relative H2DCFDA fluorescence level. Each value on the graph is the mean of three measurements and error bars show the standard deviation (SD). *p < 0.05, **p < 0.01 and ***p < 0.001 represent significant effects between treatments and control followed by a Dunnett’s test

The results of the analysis of the apoptosis level in melanoma cells showed a large decrease in the activity of caspases 3/7 in comparison with the control cells (Figs. 7and 8). This is in line with the test results for cell viability. The tested LL does not cause apoptosis in cancer cells and even inhibits it already at low concentrations. On the other hand, in healthy cells—fibroblasts, the LL tested causes a significant increase in the level of 3/7 caspase activity in the lowest concentrations analyzed. In turn, at concentrations of 2.5% and higher, caspase activity again falls below the control level, which may mean necrotic cell death due to the action of the LL tested. As apoptosis in human cells could be connected with changes in the oxidative stress level, ROS content was also measured as an indicator of oxidative stress. Obtained results are depicted in Fig. 9. In case of fibroblasts, an observed increases in ROS content are probably connected with decreases in relative cell viability. In the highest studied concentrations, the most significant increases in studied parameter were observed. Similar results were obtained for melanoma cells.

**Fig. 10:**
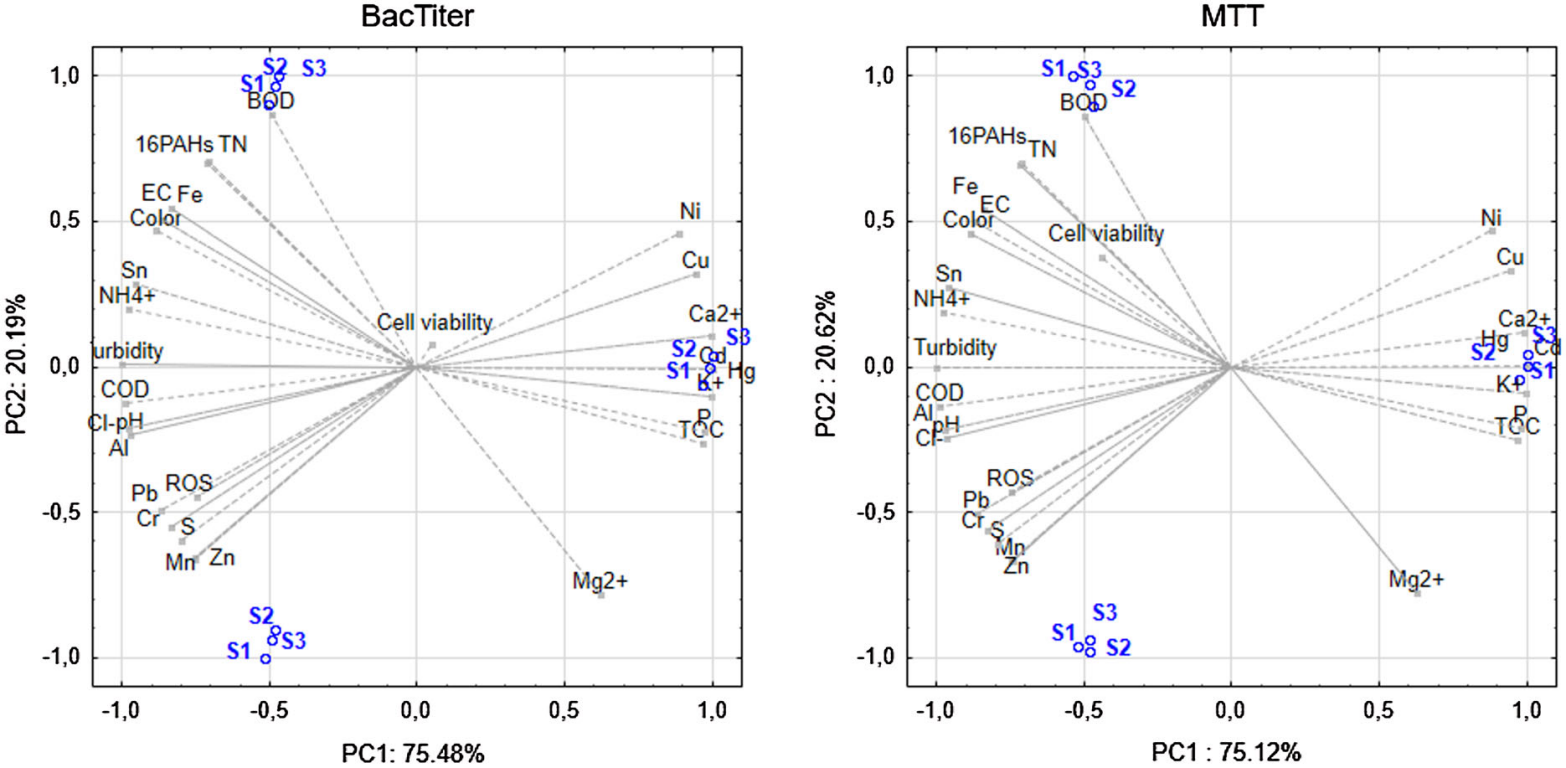
Biplot analysis for cell viability of *B. subtilis* (S1), *P. fluorescens* (S2) and *E. coli* (S3) treated with LL measured with BacTiter™ and MTT method and physicochemical properties of landfill leachates

**Fig. 11:**
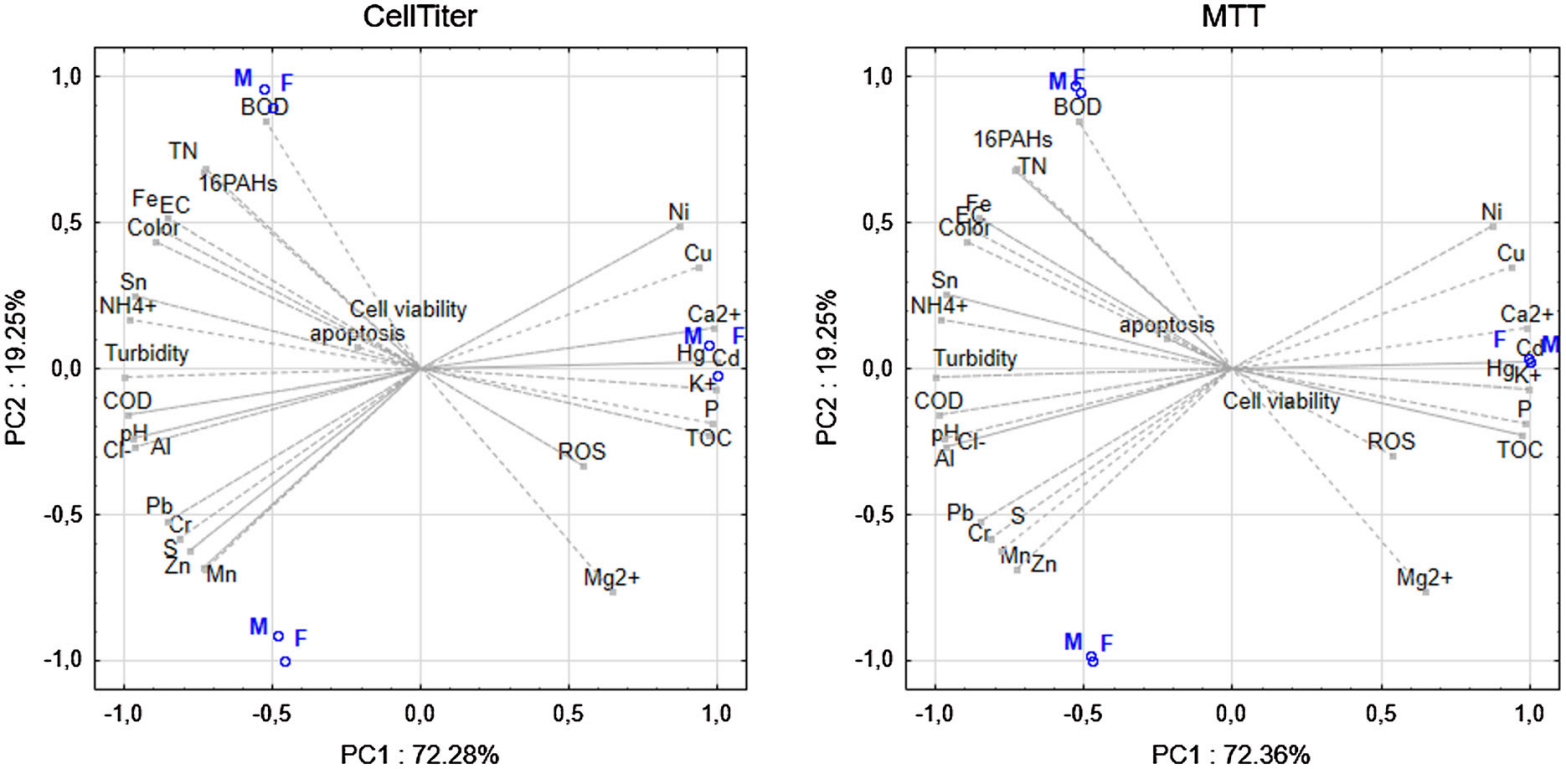
Biplot analysis for cell viability of fibroblasts (F) and melanoma (M) treated with LL measured with CellTiter^™^ and MTT method and physicochemical properties of landfill leachates

Figures 10 and 11 present relationships between physicochemical properties of LL and human cell viability, apoptosis and ROS content (A-375 melanoma and fibroblasts) and tested bacteria cells (*B. subtilis, P. fluorescens* and *E.coli*) viability and ROS level. Both, viability of studied human cell lines and bacteria cells, indicate connection with parameters such as BOD and the concentrations of Ca^2+^, Cd, Hg and K^+^. Most of the studied variables are represented by first component which explained over 70% variability. In case of human cell lines, a connection between cell viability, apoptosis and PAHs content was noticed, which may result from an evident influence of selected PAHs present in LL on cell metabolism. ROS level was correlated with the level of heavy metals such as Cd and Hg. Analyzing the data presented in Fig. 10, it can be concluded that the level of ROS in bacterial cells could be influenced by metals present in LL, such as Cr and Pb, while the viability of bacterial cells depended on the content of PAH’s (in particular using the MTT method).

## Discussion

Landfill leachate is a potentially dangerous by-product generated during the storage of various types of waste. It may contaminate soil and groundwater not only in the immediate vicinity of the landfill, but also at large distances from it, constituting a potential threat to human health. Its risk assessment may be based on physicochemical analysis of samples taken from landfills and on biological tests utilizing bacterial cultures, algae, aquatic invertebrates and fish. From about 20 years, there has been observed a steadily growing trend of using human cell cultures in vitro as a model for investigating the biological effects of exposure to the leachate (Baderna et al. 2019). However, usually, the first step in the environmental sample analysis is its physicochemical study. Results obtained from the physicochemical analysis revealed that studied leachate samples are characterized by high concentration of COD, BOD and PAHs. Due to the high content of organic matter, a large load of nitrates and ammonium nitrogen, it may pose a potential threat after entering directly into the environment without any prior treatment processes. We also observed a dark color of the LL, which is usually connected with the presence of humic substances. Presented results are consistent with the literature data indicating representation of high content of organic matter (Vedrenne et al. 2012). Qiu A et al. presented the order of toxicity of heavy metal ions, Cr > Cd > Cu > Zn > Ni indicating elements, which possess the highest toxic potential. Authors also stated that various heavy metal ions showed more than one kind of toxicity (Qiu et al. 2016). Qiu A et al. observed that even trace amounts of heavy metal ions could accumulate within the cells and eventually produce toxic effects in microorganisms. We also noticed high levels of above-mentioned metal ions, which may be responsible for an effect observed especially in human cells sensitive to extremely low levels of environmental toxins. On the other hand, bacterial cells are characterized by higher resistance to environmental pollution. An unexpected result was an increase in bacterial cell viability, which could be explained by the use of some LL compounds as a source of metabolites necessary for bacterial cells growth and development. *E. coli* and *B. subtilis* are being used for LL toxicity testing (Thomas et al. 2009). *E.coli*, as a human pathogen and one of the basic sanitary indicators, is a microorganism that successfully develops in an environment rich in carbon compounds and various minerals that are dangerous pollutants. Therefore, similarly as Chekabab et al. and van Elsas et al. indicated, we observed a significant increase in *E. coli* relative viability under the influence of LL (Chekabab et al. 2013; van Elsas et al. 2011). Our results indicate that *B. subtilis* was more sensitive to LL in concentrations below 1%. However, presented data indicating increases in relative *B. subtilis* viability as compared to control in higher LL concentrations are in accordance with data obtained by Takigami et al. indicating that *B. subtilis* is rather resilient to chemicals (Takigami et al. 2002). As it was mentioned analyzed leachates may serve as a source of nutrients to *B. subtilis*. Thomas et al. suggested that chemical resistance and specific metabolic properties make this bacterial strain an ideal candidate for LL toxicity testing (Thomas et al. 2009). Considering *E. coli* only 13% LL concentration exhibited stimulatory effect on cells viability. In case of *P. fluorescens* LL concentrations higher than 6% were cytotoxic. Both implemented methods gave similar results of bacterial cells viability, indicating rather stimulatory than cytotoxic effect. Observed changes in ROS content in bacterial strains may influence cells viability, which is in accordance with the results obtained by Rummel et al. (2019) indicating the activation of the oxidative stress response by leachate.

Although cell lines such as MCF-7, HepG2 or lymphocytes are most commonly used in this type of research, other cell lines are also utilized as a biological model for in vitro analysis of the effects of environmental contaminants on human health (Baderna et al. 2019). Examples include normal human dermal fibroblasts and human melanoma of the Me45 line, which were studied in the work of Widziewicz et al. (2012) comparing the effects of raw and treated leachate from landfills from various treatment systems. Our data also demonstrated that analyzed LL exhibits significant influence both on normal, healthy cells and cancerous cells, but it should be noted that completely different results were obtained in two different cell lines. The analyzed range of concentrations of LL to which studied cells were subjected was similar as indicated in the literature data where cytotoxic effect was observed (Ghosh et al. 2015; Widziewicz et al. 2012). Presented results indicate that significant changes in relative cell viability are noticed from 1.5 to 5% LL concentrations and it concerns both cell lines studied but with the opposite effects on analyzed parameter. Baderna et al. studied leachate cytotoxicity in similar concentration range and they found out that leachate is cytotoxic even at low concentration but after longer incubation time (Baderna et al. 2011). Our results indicating cytotoxic and cancerogenic potential of studied filtrates are in accordance with the results of chemical analysis. Leachate contains a large amount of cytotoxic and carcinogenic compounds, such as heavy metals, PAHs and ammonia, which may cause oxidative stress and thus induce apoptosis. This was also demonstrated by Eckers et al. (2009). We observed that rather lower LL concentration, in the range between 1.5 and 2%, caused significant increase in apoptosis level in fibroblasts. In melanoma cells, we did not observe any significant changes above the level of control untreated cells. Presented decreases in caspases 3/7 activity may indicate that LL inhibits apoptosis in cancer cells making them more resistant. Similarly, Alabi et al. (2013) analyzed selected parameters in fibroblasts treated with leachate in the wide range of concentrations and they observed apoptotic DNA fragmentation in cells exposed to lowest leachate concentration, while necrosis was found after treatment with higher concentrations, as 20–40%. We also confirmed our data by dual staining of cells exposed to selected concentrations of LL. All these data support the hypothesis that an apoptotic pathway may be responsible for the toxicity of leachate. The results revealed also an increase in ROS content in both human cell lines. In fibroblasts, high level of ROS is usually connected with the decrease in cell viability caused by oxidative damage of cells. Melanoma cells, as cancerous cells, are more resistant to oxidative stress and their proliferation rate is enhanced through the activity of ROS. The literature data point to the fact of the induction of oxidative stress in human cells by leachate (Gupta et al. 2019).

## Conclusion

The literature data indicate that many hazardous chemical compounds have been identified in the leachate, which raises serious concerns for the safety and health of humans and animals, affecting their ecology and food chains (Naveen et al. 2017). While discharging it directly into environment such a leachate may constitute a significant source of water and ground pollution. In this study, we presented toxicological effects of the leachate obtained from the local landfill confirmed by different chemical and biological tests. Biological part of the experiment constituted different bioassays applied in order to demonstrate a variety of responses to LL. Presented results demonstrate rather low toxicity of analyzed LL in bacterial cells and even its stimulating activity toward *E. coli*, which is a pathogenic strain. Stimulation of growth of pathogenic strains can be a serious threat to human health. Simultaneously, observed high toxicity toward human cells indicates that these leachates may pose a health hazard for exposed human populations and the whole human environment. The obtained results clearly indicating the toxicity of the tested leachates may be an excellent starting point for further studies of the mechanisms of leachate toxicity in various biological models. They also indicate the need to monitor the presence of even trace amounts of leachate in the environment, which is released from the landfill into the human environment in an uncontrolled manner.
